# Net Promoter Score Model for Evaluating Paediatric Medicine Acceptability: Validation and Feasibility Study

**DOI:** 10.3390/pharmaceutics16121513

**Published:** 2024-11-25

**Authors:** Okhee Yoo, Demi Stanford, Britta S. von Ungern-Sternberg, Lee Yong Lim

**Affiliations:** 1 Division of Pharmacy, School of Allied Health, The University of Western Australia, Perth, WA 6009, Australia; okhee.yoo@uwa.edu.au (O.Y.); 23433436@student.uwa.edu.au (D.S.); 2 Institute for Paediatric Perioperative Excellence, The University of Western Australia, Perth, WA 6009, Australia; britta.regli-vonungern@health.wa.gov.au; 3 Perioperative Medicine Team, Perioperative Care Program, The Kids Research Institute, Perth, WA 6009, Australia; 4 Division of Emergency Medicine, Anaesthesia and Pain Medicine, Medical School, The University of Western Australia, Perth, WA 6009, Australia; 5 Department of Anaesthesia and Pain Medicine, Perth Children’s Hospital, Perth, WA 6009, Australia

**Keywords:** medicine acceptability, paediatric drug development, palatability assessment, medicine acceptability score, taste-masked formulations, pharmaceutical formulation optimisation, patient adherence, taste assessment

## Abstract

**Background/Objectives**: Medicine acceptability is crucial for paediatric drug development, yet its assessment remains challenging due to the multifaceted nature of sensory attributes like taste, smell, and mouthfeel. Traditional methods of acceptability evaluation often involve complex questionnaires and lack standardisation, leading to difficulties in a comparative analysis across studies. This study aimed to develop a simplified, standardised approach for assessing medicine acceptability introducing the Net Promoter Score (NPS) framework to derive a Medicine Acceptability Score (MAS). **Methods**: A retrospective analysis was conducted using taste assessment data from nine paediatric formulations across four studies. The MAS was calculated by identifying an optimal range for categorising participant responses, which encapsulated diverse sensory attributes into a single metric. Validation was performed across various age groups and different formulations to test the reliability and discriminatory power of MAS. **Results**: The MAS effectively discriminated between acceptable and unacceptable formulations, providing a practical tool for formulation development. **Conclusions**: The MAS offers a novel, standardised metric for evaluating paediatric medicine acceptability, addressing key limitations of traditional methods. Future studies are recommended to refine the MAS model through the establishment of benchmark scores for chronic and acute medications, thereby standardising acceptability assessment of medicines across the pharmaceutical industry.

## 1. Introduction

Medicine acceptability refers to the capacity and readiness of both patients and their caregivers to appropriately use the medicine as prescribed [1]. Measurement of medicine acceptability has gained traction in paediatric medicine development in recent years, and its accurate measurement is regarded to be important to guide formulation optimisation decisions [2,3,4]. Medicine acceptability is multifactorial, as it requires the various elements of the medicinal dosage form and its sensory attributes meeting the users’ expectations [2,3]. The challenge in medicine acceptability measurement is in finding a suitable model to distil these critical elements into a single quantitative score that is not only a true reflection of medicine acceptance but is easy to apply and reproducibly measured across different test sites [3,5].

In the Quality by Design approach to medicine development, the critical attributes of the Quality Target Product Profile (QTPP) are predefined. The dosage form for the target product is often predetermined and, for paediatric oral medicines, the desirable dosage form may be a liquid, a chewable tablet, a minitablet, or an orally disintegrating tablet that allows for dose flexibility and ease of swallowability across the paediatric age range [6,7]. The development process is then aimed at optimising the formulation and manufacture process to yield a product that best meets the predefined QTPP criteria. A critical QTPP attribute underscoring the acceptability of an oral paediatric medicine is its palatability, often regarded to be an all-encompassing marker of sensory attributes including smell, taste, mouthfeel, and effectiveness of masking of aversive drug taste [8]. The gold standard for palatability evaluation is taste scores provided by a human taste panel. In the absence of regulatory guidelines, however, the taste scores obtained for a new formulation and its comparator are frequently compared by statistical methods, with arbitrarily applied criteria and a cutoff used to conclude whether the new formulation is acceptable or not to the target population [9,10,11]. Study designs for palatability evaluation lack harmonisation, with laboratories adopting a range of food industry sensory evaluation tools that include hedonic scales, visual analogue scales, and category scales [9,10,11,12,13]. Some evaluations considered single sensory traits, e.g., bitterness taste, while others measured multiple characteristics, e.g., grittiness and taste [13,14]. This diversity results in rich data but makes it difficult to conduct interstudy comparisons of different formulations of the same drug. Attempts have been made to standardise medicine acceptability testing; an example is the CAST methodology [15], which generates an acceptability map from a multivariate analysis of a large data set mined from studies involving paediatric patients. This map was made available to other researchers to assess the acceptability of medicines in children [16]. However, the CAST methodology deploys an extensive, multidimensional questionnaire, which can be challenging to use on very young participants. It is also not easily applied in a medicine development process that employs the Design of Experiments method to prepare multiple formulation samples for comparative evaluation.

**Figure 1:**
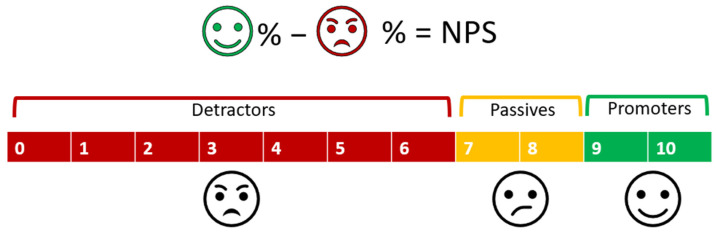
An illustration of how the Net Promoter Score (NPS) is calculated. The NPS ranges from −100 to +100 and is calculated by subtracting the percentage of detractors (customers who give a score of 0–6) from the percentage of promoters (customers who give a score of 9–10).

A novel approach proposed by our laboratory for the evaluation of medicine acceptability involves the Net Promoter Score (NPS) widely applied by businesses to assess customer experience, predict sales growth, and guide management decisions [17]. The NPS is a contemporary metric designed to streamline the customer feedback process by addressing the biases inherent in earlier methods, specifically the “yea saying” and “nay saying”, which reflect the tendency of individuals to agree or disagree with questionnaire items without regard to their content. These biases can significantly skew the conclusion of a study. The NPS model deploys a single-question survey, asking participants to respond to the question “How likely is it that you would recommend [Company X/Product X] to a friend or colleague?” using an eleven-point scale, which ranges from zero (not at all likely) to ten (extremely likely). Participants are categorised into promoters (score 9–10), passively satisfied (7–8), and detractors (0–6), and the NPS is calculated by subtracting the percentage of detractors from the percentage of promoters (Figure 1).

Our hypothesis is that the NPS principles may be applied to simplify medicine acceptability evaluation by encapsulating the multiple factors influencing a child’s willingness to take a medicine into a single, comprehensive Medicine Acceptability Score (MAS) [17]. The hypothesis is yet to be tested even though the NPS model has been applied to measure patient satisfaction in the healthcare sector. The aim of this study was to determine the feasibility of applying the NPS model to the evaluation of taste-masked paediatric formulations developed and tested for palatability in our laboratory. The objective was to establish a validation methodology to assess the feasibility and clarity of applying the NPS approach to generate a MAS that would correlate to participants’ willingness to take the test formulation again when unwell.

## 2. Materials and Methods

### 2.1. Data for Generating the Medicine Acceptability Score

This is a retrospective study employing data collected for 9 medicinal formulations in 4 taste assessment studies conducted by our laboratory. Studies 1, 2, and 4 have been published [9,10,11] while Study 3 was reported in the PhD thesis of first author Yoo [18]. Studies 1 and 2 were clinical trials approved by the Human Research Ethics Committees of the Western Australian Child and Adolescent Health Service (2014102EP, ACTRN12615000225516; and RGS0000000205, ACTRN1261700149369, respectively). Studies 3 and 4 were preclinical studies approved by the Human Research Ethics Committee of the University of Western Australia (UWA) (RA/4/20/5753 and ET000155, respectively). The test formulations were novel oral paediatric formulations developed by our laboratory and their respective comparators. Signed written consent was provided by the guardians of the child participants (Studies 1 and 2) or by the adult participants themselves (Studies 3 and 4). The 4 studies are summarised in Table 1.

Study 1 was a prospective, open-label, single-centre, randomised, single-treatment clinical trial conducted at Princess Margaret Hospital (now Perth Children’s Hospital) in Perth, Australia, to compare the palatability of a novel midazolam taste-masked tablet (MDM TMT, 5 mg midazolam) and an oral liquid comparator (MDM LQD, 5 mg/mL) [10]. Participants were children aged 4–16 years scheduled for surgery and prescribed midazolam as premedication. Each child participant evaluated only one formulation and was randomly assigned to receive MDM TMT or MDM LQD at a midazolam dose of 0.5 mg/kg. The administered midazolam doses ranged from 6.25 to 20 mg, which corresponded to 1¼ to 4 tablets of MDM TMT or 1.25 to 4 mL of MDM LQD. The participants were asked to score how much they like the taste of the assigned formulation using a 5-point hedonic scale and to provide a ‘Yes’ or ‘No’ answer to a question on their willingness to take the formulation again if they were sick. The responses of 74 participants were recorded for MDM TMT and the responses of 70 participants were recorded for MDM LQD.

Study 2 was a pilot, single-centre, open-label, randomised, single-treatment clinical study conducted at the Perth Children’s Hospital to compare the palatability of a novel tramadol taste-masked tablet (TRM TMT, 10 mg tramadol) and its oral liquid comparator (TRM LQD, 5 mg/mL) [9]. Children aged 3–16 years scheduled for surgery and prescribed tramadol were recruited, with 68 participants assigned to receive TRM TMT while 71 participants received TRM LQD. Each participant was assigned to evaluate only one tramadol formulation and was asked to score how much they like the taste of the assigned formulation using a 5-point hedonic scale. The tramadol dose was 1 mg/kg, and the administered dose per participant ranged from 12.5 to 80 mg, which corresponded to 1¼ to 8 tablets of TRM TMT or 2.5 to 16 mL of TRM LQD. The participants also provided a ‘Yes’ or ‘No’ answer to a question on their willingness to take the formulation again if they were sick.

**Figure 2:**
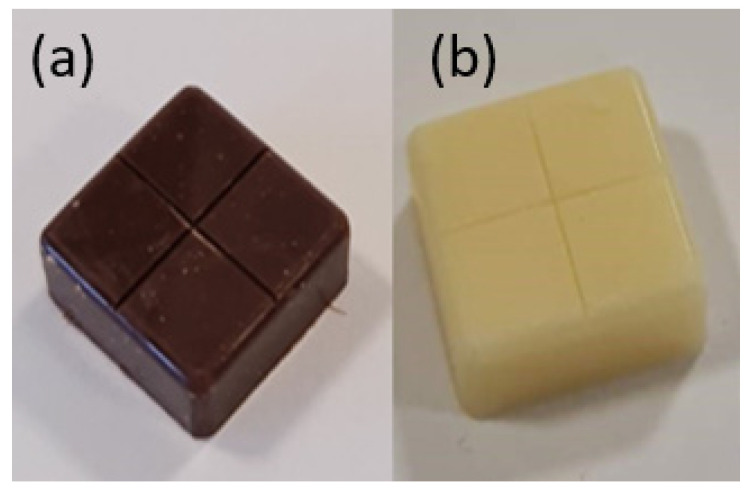
Chewable flucloxacillin taste-masked tablets equivalent to 62.5 mg flucloxacillin and with (**a**) dark chocolate as carrier (FLX TMT1) and (**b**) white chocolate as carrier (FLX TMT2).

Study 3 was a pilot, single-centre, open-label, randomised study conducted at UWA to compare the palatability of two novel taste-masked tablets of flucloxacillin (FLX TMT1 (Figure 2a) and FLX TMT2 (Figure 2b), each containing 62.5 mg flucloxacillin) against Flucil™, a commercial flucloxacillin suspension (FLX LQD, 250 mg/mL) as a comparator. The participants (*n* = 21) were healthy young adult volunteers aged 18 to 25 years recruited from the UWA community who passed a pre-screening test on taste perception. Unlike Studies 1 and 2, Study 3 was a repeated measures study in which every participant evaluated all three FLX formulations at the same FLX dose of 62.5 mg, which corresponded to 1 tablet of FLX TMT1, 1 tablet of FLX TMT2, or 0.25 mL of FLX LQD. The sequence for testing the three formulations was randomised for the participants, and a washout period of 10–15 min was implemented between formulations. Participants used separate 5-point hedonic scales to score how much they liked the taste of each formulation and they answered ‘1’, ‘2’, or ‘3’ to a question to indicate which one of the 3 FLX formulations they were most willing to take if sick.

**Figure 3:**
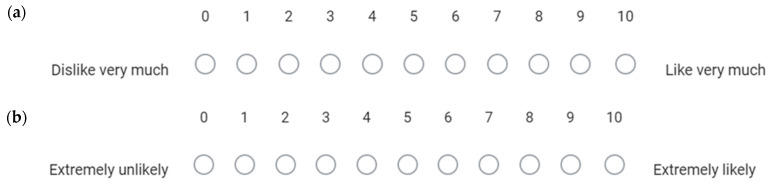
The 11-point rating scales used by the participants in Study 4 to assess each prednisolone sodium phosphate formulation for (**a**) their liking of the taste and (**b**) their willingness to take the formulation again.

Study 4 was a pilot, single-centre, open-label, randomised study conducted at UWA to compare the palatability of a prednisolone sodium phosphate taste-masked tablet (PSP TMT, 13.44 mg prednisolone sodium phosphate) against Redipred™, an oral commercial solution (PSP LDQ, 6.72 mg/mL) [11]. The participants (*n* = 25) were healthy young adult volunteers aged 18 to 25 years recruited from the UWA community. Study 4 was also a repeated measures study in which every participant evaluated both PSP TMT and PSP LQD at a fixed PSP dose of 6.72 mg, equivalent to ½ tablet of PSP TMT or 1 mL of PSP LQD. The sequence for evaluating the two formulations was randomised, and there was a washout period of 10 min between formulations. Participants rated their liking for each formulation using an 11-point rating scale (Figure 3a). They also indicated on a separate 11-point rating scale their willingness to take the formulation again if needed (Figure 3b). Following the evaluation of both formulations, the participants answered ‘1’ or ‘2’ to a third question to indicate which one of the two formulations they were more willing to take when feeling unwell.

### 2.2. Calculation of Medicine Acceptability Score (MAS) and Willingness to Take Medicine Score (WTMS) for Studies 1, 2, and 3

**Figure 4:**
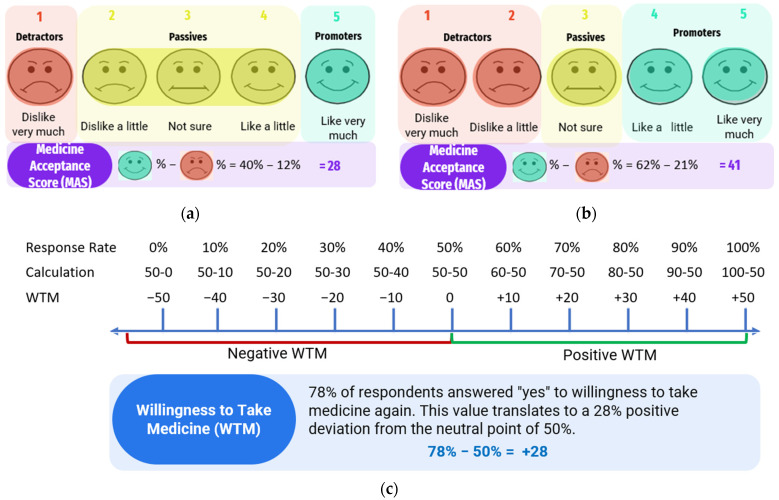
A schematic diagram illustrating how the MAS was calculated using data from a 5-Point Hedonic Taste Score and assigning the passives scores to be (**a**) 2–4; and (**b**) 3 alone, as well as the calculation of the Willingness to Take Medicine Score (WTMS) (**c**). Both MAS and WTMS were calculated using data obtained from a paediatric clinical trial on the tramadol taste-masked tablet (*n* = 68).

Participants in Studies 1, 2, and 3 provided taste scores for a total of seven medicinal formulations. For each formulation, the taste data were used to group the participants into promoters, passives, and detractors, and the Medicine Acceptability Score (MAS) was calculated by deducting the percentage of detractors from the percentage of promoters. Depending on how well the formulation had been received, the calculated MAS might be a positive or negative value. To categorise the participants into promoters, passives, and detractors, the score range for the passives had to be predefined. Figure 4 graphically explains these calculations using the TRM TMT data and the defined passives score range of 2–4 (Figure 4a) and 3 alone (Figure 4b), showing how the percentages of promoters and detractors, and ultimately the MAS value, were influenced by the defined score range for the passives.

The Willingness to Take Medicine Score (WTMS) for each medicinal formulation was calculated using data that participants provided to indicate their willingness to take the formulation again if unwell. For Studies 1 and 2, where each participant assessed only one test formulation, this question was answered with a ‘Yes’ or ‘No’ answer. Participants who provided the affirmative ‘Yes’ answer were calculated as a percentage, and since only ‘Yes’ or ‘No’ responses were evaluated, a 50% response rate was considered to represent a neutral ‘no preference’ response. WTMS for a test formulation was calculated based on how much the ‘Yes’ response rate deviated from 50%, with the WTMS recorded as a positive value if the ‘Yes’ response rate exceeded 50% and as a negative value if the ‘Yes’ response rate was below 50%. The calculation of WTMS for TRM TMT is illustrated in Figure 4c. For Study 3, where each participant tested three FLX formulations, the participants provided answers of ‘1’, ‘2’, or ‘3’ to the question on which one of the three formulations they would most prefer to take if unwell. The number of affirmative responses for each FLX formulation was calculated as a percentage and, because Study 3 was a comparison of 3 formulations, a 33% response rate was considered to represent a neutral ‘no preference’ response. The WTMS for each FLX formulation was calculated based on how much the percentage of affirmative response deviated from 33%, with the WTMS recorded as a positive value if the response rate exceeded 33% and as a negative value if the response rate was below 33%.

As this study represented the first time the NPS approach was applied to medicine acceptability evaluation, it was necessary to determine the optimum passives score range to be applied for calculating the MAS. The participants in Studies 1, 2, and 3 had used the 5-point hedonic scale to provide the taste data for each medicinal formulation; therefore, 4 passives score ranges, namely 2–3, 2–4, 3, and 3–4, were applied to categorise the participants into promoters, passives, and detractors. The corresponding MAS value for each passive score range was calculated, and the MAS values were compared with the calculated WTMS values. The passives score range that yielded MAS values that most closely matched the WTMS values was determined to be the optimal passives score range, and this was applied for the validation study conducted using data from Study 4.

### 2.3. Validation

Each participant in Study 4 evaluated two PSP formulations (PSP TMT; PSP LQD) and provided responses to three questions for each formulation: how they like the taste of the formulation, which was rated on an 11-point scale; their willingness to take the formulation again if unwell, which was rated on another 11-point scale; and finally, which of the two PSP formulations they were more willing to take again if unwell, which required a ‘1’ or ‘2’ answer. For each formulation, two MAS values were calculated; the first MAS was based on the ‘taste’ scores, and the second MAS was based on ‘willingness to take the formulation again’ scores. To calculate both MASs, the optimal passives score range obtained from the collective data of Studies 1, 2, and 3 was applied. However, as the 5-point hedonic scale was used in Studies 1, 2, and 3 whereas the 11-point scale was used in Study 4, we adjusted the optimal passives score range by multiplying by 2 before applying it to calculate the MAS in Study 4. Scaling of the optimal passives score range of 3 resulted in a value of 6, and the passives score range of 5 to 7 was applied to Study 4. For each PSP formulation, the number of affirmative answers received in response to the 3rd question was calculated as a percentage and the WTMS was calculated by comparing this percentage to 50%, which represented a neutral ‘no preference’ response as Study 4 was a comparison of two formulations. Validation was considered to be achieved when the WTMS correlated to the MAS for the formulation.

To validate the findings, an additional analysis was conducted for Studies 1 and 2, in which the participants were divided into two age groups: young (≤6 years) and mature (>6 years). For each group, the MAS was calculated using the selected passive score of 3 and then compared with the WTMS scores.

## 3. Results

**Figure 5:**
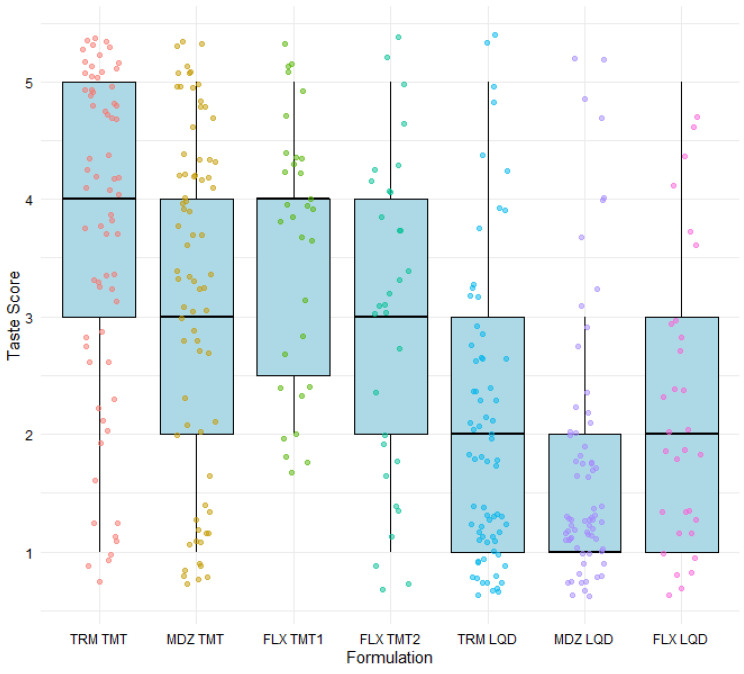
Box plots showing the taste scores obtained via 5-point hedonic scales for medicinal formulations evaluated in Studies 1–3. The upper limit of each box represents the 75th percentile, the lower limit represents the 25th percentile, and the line within the box indicates the median value. Whiskers above and below the boxes represent the 90th and 10th percentiles, respectively. Individual data points are depicted as solitary circles. Tramadol = TRM; Midazolam = MDZ; Flucloxacillin = FLX; Taste-masked tablets = TMTs; LQD = oral liquid comparator. Two flucloxacillin taste-masked tablets, FLX TMT1 and FLX TMT2, were evaluated.

Taste scores provided by participants using similar five-point hedonic scales for the seven medicinal formulations evaluated in Studies 1, 2, and 3 are provided in Figure 5. The raw data exhibited wide variability in taste scores, with extreme dislikes or likes significantly affecting the mean taste score. For the taste-masked tablets, TRM TMT and FLX TMT2 had high median scores of 4 while MDZ TMT and FLX TMT1 both had median scores of 3 (Table 2). The liquid comparators had lower median scores than the corresponding taste-masked tablets, with MDZ LQD showing the lowest median score of 1. Table 2 shows the affirmative responses of participants on their willingness to take the test formulation again when unwell. While there was agreement between the willingness to retake response and the taste scores for TRM TMT, showing this formulation to be the most favoured of the seven tested formulations, the percentage of affirmative responses did not quite correlate to the taste scores for the other formulations. As an example, although MDZ TMT had lower taste scores than FLX TMT1, the percentage of participants willing to retake MDZ TMT was higher compared to the percentage willing to retake FLX TMT1. Additionally, the percentage of participants willing to retake MDZ LQD, which had the lowest taste scores, was 7-fold higher than the percentage willing to retake FLX LQD. Based on the willingness to retake response, FLX LQD would rate as the least favoured formulation, even though its median taste score was comparable to TRM LQD and higher than MDX LQD.

**Figure 6:**
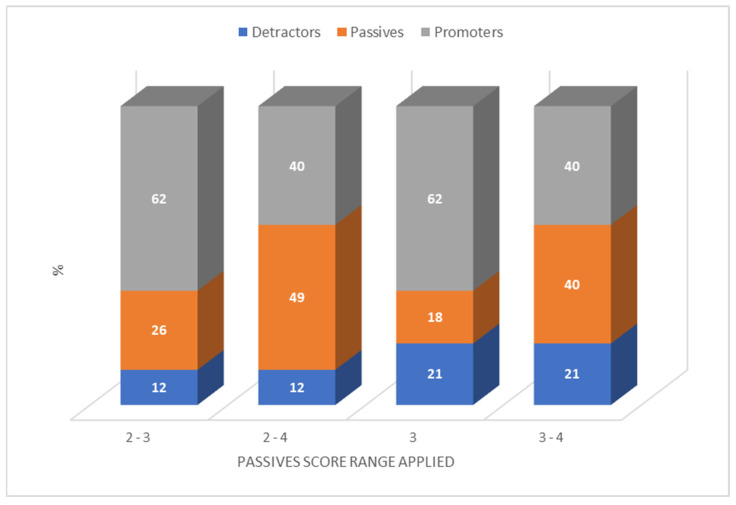
Percentages of promoters, passives, and detractors for TRM TMT when different score ranges were defined for the passives category. Participants were categorised into promoters, passives, or detractors based on the taste scores they provided for TRM TMT using a 5-point hedonic scale.

MASs was calculated by categorising the participants in Studies 1, 2, and 3 as promoters, passives, and detractors based on the taste scores they provided. Figure 6 shows how the percentages of promoters and detractors shifted for TRM TMT when the different passives score ranges of 2–3, 2–4, 3, and 3–4 were applied. These shifts were observed for all seven medicinal formulations; consequently, different MAS values were obtained for each formulation depending on the passive score range applied (Table 3). WTMS for each formulation was also calculated based on the percentage of participants willing to retake the formulations when unwell (Table 3).

All the taste-masked TMT formulations had positive WTMS whereas all the LQD comparators had negative WTMS. The ranking order for WTMS was TRM TMT > FLX TMT1 > MDZ TMT ~ FLX TMT2 > MDZ LQD > TRM LQD > FLX LQD. None of the applied passives score ranges yielded MAS values that corresponded to this ranking order for the seven formulations. However, for the LQD comparators, high negative MAS values were obtained across all four passives score ranges applied, which unambiguously confirmed that these formulations are ‘unacceptable’. For the TMT formulations, a mix of positive and negative MASs were obtained depending on the passives boundaries applied. Positive MAS values (>0) were obtained for all TMT formulations only when the assigned passives scores were 2–3 and 3, and not when the passives score ranges were 2–4 and 3–4. This suggests that the passives score ranges of 2–4 and 3–4 were inappropriate for achieving the correlation of MAS and WTMS for the TMT formulations. The further comparison of the passives ranges of 2–3 and 3 for the TMT formulations indicates that the ranking order of MAS calculated using the passives score range of 3 most closely matched that of the WTMS. The optimum passives score range was therefore considered to be 3.

**Figure 7:**
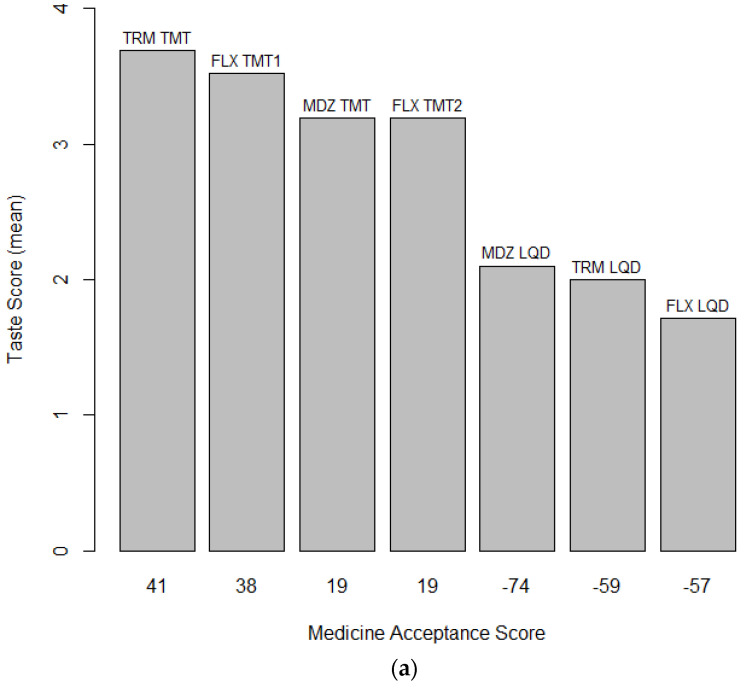

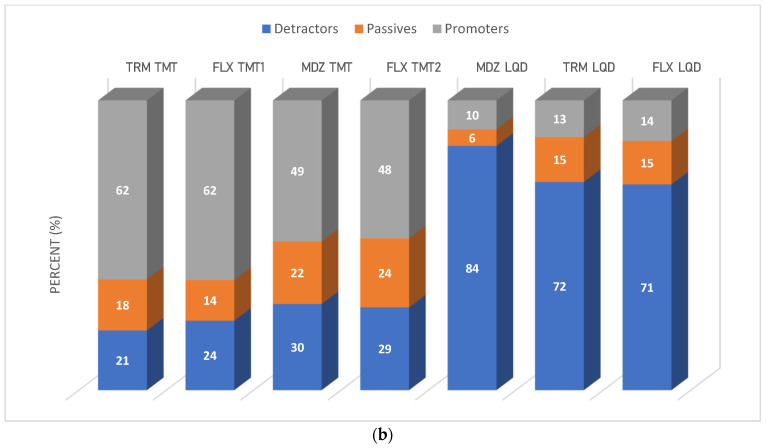
(**a**) The bar graph depicts the correlation between mean taste scores with a passives range of 3 and Medicine Acceptance Scores (MAS) for 7 medicinal formulations evaluated in Studies 1–3. Formulations with positive MAS values are grouped on the left, indicating higher acceptance, while those with negative MAS values are on the right, indicating lower acceptance. The data illustrate how MAS can differentiate acceptance levels that are not apparent from comparing taste scores alone. (**b**) The percentages of promoters, passives, and detractors for the different formulations. Participants were categorised as promoters, passives, or detractors based on their taste scores using a 5-point hedonic scale, with a score of 3 indicating a passive response. Tramadol = TRM; Midazolam = MDZ; Flucloxacillin = FLX; Taste-masked tablets = TMTs; LQD = oral liquid comparator.

Figure 7a shows the mean taste scores plotted against MAS calculated using the optimum passives score range of 3 for the seven formulations. Two clusters become apparent; the TMT formulations with mean taste scores higher than 3 all showed positive MAS (range 19 to 41) whereas the comparator LQD formulations with mean taste scores of 2 or lower all had negative MAS (range: −74 to −57). Figure 7b shows the percentage of promoters, passives, and detractors obtained by applying the optimum passive score range of 3 for the seven formulations. There are again two clusters, the LDQ formulations with negative MAS attracting more than 70% detractors and the TMT formulations with positive MAS attracting at least 48% promoters. This graphically illustrates how the MAS is a simple but effective tool for differentiating medicinal acceptability compared to arbitrarily assigning a cutoff to conclude whether the difference in mean taste score between a test formulation and its comparator is clinically significant.

**Figure 8:**
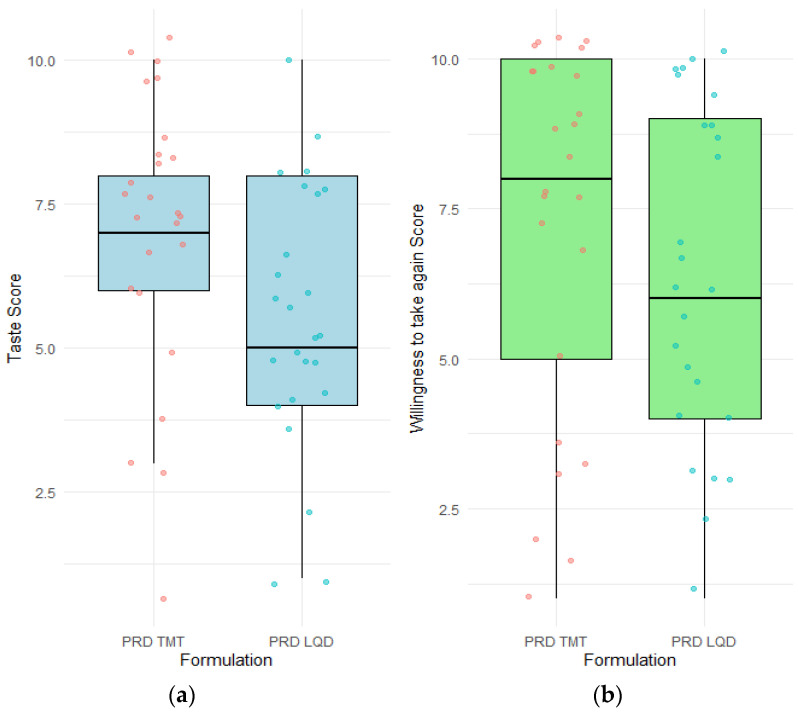
Box plots showing the scores obtained via 11-point scales for (**a**) taste; and (**b**) willingness to take the formulation again when unwell for two prednisolone sodium phosphate formulations in Study 4. The upper limit of each box represents the 75th percentile, the lower limit represents the 25th percentile, and the line within the box indicates the median value. Whiskers above and below the boxes represent the 90th and 10th percentiles, respectively. Individual data points are depicted as solitary circles. Prednisolone sodium phosphate = PSP; Taste-masked tablet = TMT; LQD = oral liquid comparator.

Participants in Study 4 evaluated PSP TMT and PSP LQD by providing the taste score for each formulation on an 11-point scale and recording their willingness to take the formulation again when sick on another 11-point scale. These scores are provided in Figure 8. PSP TMT had higher median taste scores and a higher median ‘willingness to retake’ score than PSP LQD. To the third question asking participants to decide on which one of the two PSP formulations they were more willing to take again when unwell, 56% of the participants chose PSP TMT over PSP LQD (Table 4). The higher percentage correlated well with the higher median taste and ‘willingness to retake’ scores for the PSP TMT (Table 4).

Two MASs were calculated for each PSP formulation using the taste scores and the ‘willingness to retake’ scores, respectively. The optimal passives score range of 3 derived using the 5-point hedonic scale in Studies 1–3 was transposed to the 11-point scale and the passives score range of 5–7 was applied to calculate the two MASs for Study 4. MAS calculated based on taste scores was +32 for PSP TMT and 0 for PSP LQD, whereas MAS calculated based on ‘willingness to retake’ scores showed positive values for both PSP TMT and PSP LQD (Table 5). WTMS was also calculated, with PSP TMT showing a positive WTMS and PSP LQD having a negative WTMS. Thus, the WTMS for the PSP formulations showed better correlation to the MAS calculated using the taste scores than to the MAS calculated using the ‘willingness to retake’ scores (Table 5).

When data were stratified into young (≤6 years) and mature (>6 years) age groups, all LQD formulations showed a negative WTMS when the MAS was negative (Table 6). When the WTMS was positive, MAS values were either positive or zero. For MDZ TMT formulations, young participants tended to have higher MAS values and lower WTMS, whereas mature participants showed lower MAS and higher WTMS values. This may suggest that, in this setting, the mature participants provided more rational responses in terms of willingness to comply when needed, compared to younger participants. A similar trend was observed for MDZ LQD formulations, although this trend was less pronounced with both TRM formulations, possibly due to differences in the testing environment between MDZ and TRM.

## 4. Discussion

Medicine acceptability evaluations ranged in complexity, and while a comprehensive study requiring extensive profiling of the medicine [19] can be helpful, a simpler approach is preferred during the medicine development phase where multiple trial formulations have to be evaluated to arrive at an optimised formulation. The MAS model developed in this study is an example of a simpler approach for assessing medicine acceptability.

The wide distribution of responses is inherent in the data we gathered on medicine taste acceptability. The same medicine can elicit a range of extreme responses, a trend we have observed across multiple studies. This variation is likely due to individual differences in taste perception, as the unique composition of each person’s taste receptors can lead to diverse flavour experiences.

Given this variability, the question arises: how should we represent medicine acceptability? Should we use the median, or mean, or a qualitative assessment? We believe the focus should be on counting the number of participants willing to accept the medicine, rather than the intensity of their willingness to take the medicine. As an example, consider a scenario in which five participants provide scores of 1, 3, 3, 4, and 4 on a 5-point hedonic scale, where 5 represents “like very much”, 3 represents “not sure”, and 1 represents “dislike very much”. In this example, one participant provides a negative score, two provide positive scores, and two are undecided (neutral scores). The mean (3) and median (3) indicate neutrality, while the MAS of 20 suggests positivity. This is because the MAS emphasises the counting of positive and negative responses, and excluding undecided scores, from the measure of acceptability. In contrast, the mean takes into account both the intensity of willingness and unwillingness, and the median provides a neutral point without reflecting on the distribution of scores on either side. Furthermore, measures like mean and median require additional values, such as standard deviation or quartiles, to describe the score distribution effectively. A qualitative assessment, while potentially insightful, demands a more complex approach, such as taste profiling, which may be less practical during the early stages of medicine development.

The advantage of using MAS lies in its simplicity, with its focus on the number of participants willing to accept the medicine, rather than the degree of their willingness. Ultimately, our goal is to determine how many people would accept a medicine formulation from the wide range of formulations that would be prepared in a typical DoE setup, and the MAS offers a straightforward metric for the pharmaceutical industry to assess this acceptability.

Reichheld, when developing the NPS approach for assessing business performance, identified that the most appropriate question to ask participants was, “How likely is it that you would recommend [Company X/Product X] to a friend or colleague?” [20]. In this study, we have shown that medicine acceptability could be assessed using the taste scores collected for the medicinal formulation and applying an appropriate passives score range to calculate the MAS. The MAS method was applied to diverse taste data collected for nine formulations of four drugs from different participant demographics and environments. Studies 1 and 2 involved children scheduled for surgery in hospital settings while Studies 3 and 4 employed healthy young adult volunteers in a university. In Study 4, the MAS was also determined using the ‘willingness to retake’ score; however, the MAS calculated using taste scores showed better correlation with WTMS, probably because the passives score range was also determined using the taste scores. If the passives score range was determined using another measured parameter, e.g., ‘willingness to retake’ scores, the resultant MAS might well be highly correlated with that parameter. Further studies employing more extensive data sets would be helpful to provide further insights into the application of taste scores to calculate the MAS.

The taste data in Studies 1–3 had a narrow range from 1 to 5 because they were collected using 5-point scales, whereas the calculated MAS can range from −100 to 100. A more granular scale that provides a greater number of response categories or cutoff points could provide more useful taste scores; however, children under seven might struggle to understand and use the more granular scales [21]. Even the five-point hedonic scale was found by some parents and caregivers to be too difficult for very young children to provide the taste scores in our Studies 1 and 2. Scoring using the 5-point scale provided only a 0.5-point difference between the lowest and highest mean taste scores for the four taste-masked TMT formulations in Studies 1–3, whereas the calculated MAS for the formulations ranged from 19 to 41. The wider spread of MAS values could potentially allow for a more nuanced decision to be made; e.g., a threshold MAS value could be imposed to further discriminate between several test formulations that all scored positive MAS.

The three oral liquid comparators in Studies 1–3 all had negative MAS over the four passives score ranges applied, suggesting that the MAS was particularly sensitive at differentiating formulations with unacceptable taste profiles. MAS values for these liquid formulations ranged from −74 to −57 while the difference in their mean taste scores was less than 1, and the difference in their mean scores from those of TMT formulations was less than 2. MDZ LQD and TRM LQD were products compounded by the hospital pharmacy without added flavouring agents, and the poor taste of these liquids was reflected in their high negative MAS values of −74 and −59, respectively. FLX LQD was Flucil™, a commercial paediatric oral formulation with several flavouring and sweetening agents [22]. Despite the taste-masking strategies, Flucil™ had MAS of −57, which underscores its notorious poor taste; doctors would avoid prescribing Flucil™ to paediatric patients to minimise therapy non-compliance [23,24]. In contrast, Redipred™, the commercial liquid comparator used in Study 4, is not known to be as foul tasting [25]. MAS for Redipred™ was 0, further highlighting how the MAS could be used to discriminate taste acceptability of medicinal formulations. The NPS model is not without critics; its reductionist approach is perceived to oversimplify complex customer behaviours and experiences that may be more effectively captured using comprehensive surveys or metrics [26]. A similar criticism could be levelled at the MAS approach, which attempts to use a simple metric to measure medicine acceptability, a multidimensional concept affected by multiple factors. In the food industry, the assessment of product acceptability is accomplished through taste trials involving expert panels able to provide a complex profiling of the sensory qualities of the test products [27]. Medicines are, however, not to be considered in the same category as food products. A medicine is prescribed for a specific therapeutic purpose, and the taste of an oral medicine needs only to be adequately acceptable for patients to willingly take the medicine again as prescribed. It would indeed not be desirable for a paediatric oral medicine to have overly attractive sensory attributes as to entice a child to take the medicine for non-therapeutic reasons. On this basis, the MAS can provide a simple and adequate tool to guide decisions on whether a test formulation may be acceptable or rejectable during the medicine development process.

Another criticism of the NPS model is its focus on extremes. It emphasises promoters and detractors while largely ignoring the passives group, a significant portion of the customer base who might provide valuable feedback and insights into how they could be converted into promoters [28]. However, it could be difficult to capture feedback from the passives respondents when assessing objective data for the multiple test formulations developed during the medicine formulation optimisation process. This feedback could be better obtained through focus groups as part of the consumer engagement process for the formulation development.

A limitation in this study is that the MAS was correlated only to WTMS calculated using the participants’ reflections of their willingness to retake the formulation. Other user-relevant measures (e.g., medication compliance) for calculating WTMS have not been explored. WTMS, obtained to correlate with MAS, was based on retrospective data. The limitation lies in the consistency of WTMS derived from these retrospective data, as some participants were patients in a hospital environment, potentially vulnerable to responding affirmatively to a willingness to take the medication, whereas healthy volunteers might have based their responses entirely on formulation factors. This study represents the first exploratory and feasibility assessment for using the MAS to evaluate medicine acceptability during the development phase, and a more structured prospective study may be required in the future to further validate the correlation of MAS and WTMS data. Another limitation is that, unlike the MAS, the upper limit of the WTMS was constrained by the choice of the neutral point and would not reach 100%. The upper limit of WTMS was also constrained by differences in the study designs, with Studies 1 and 2 applying the same degree of freedom to calculate WTMS whereas the WTMSs in Studies 3 and 4 were calculated using different degrees of freedom. However, the WTMS in Studies 1, 2, and 3 could still be collectively used to identify an appropriate passives score range, demonstrating the feasibility of MAS as a practical acceptability score.

Future work is needed to define the MAS range for ‘acceptable’ medicines. This requires the creation of benchmark scores and control scores, with positive ‘acceptable’ controls established for medicines prescribed for chronic and acute medical conditions. A positive control for medicines requiring a multiple-dosing regimen for chronic conditions may be required to have a non-objectionable taste and be generally well tolerated. For medicines used to treat acute conditions that do not necessitate completing a full course, the taste requirements for a positive control may be less stringent compared to those for chronic conditions. Having the positive controls will facilitate the standardisation of the MAS approach and ensure that approved formulations meet acceptable standards of acceptability. Future studies could also establish the statistical power associated with the number of participants recruited to validate the MAS approach. Larger sample sizes are preferable to help establish more robust validation methodologies.

## 5. Conclusions

In conclusion, the MAS offers a simplified method for evaluating the acceptability of medicinal formulations during the formulation developmental process. The MAS is a straightforward approach with capacity to discriminate between acceptable and unacceptable medicinal formulations. Future work should focus on creating benchmark scores and control scores to establish acceptable MAS ranges for medicines prescribed for chronic and acute medical conditions.

## Figures and Tables

**Table 1:** Summary of Studies Evaluating Palatability of Different Medicinal Formulations. TMT = novel taste-masked chewable tablet; LQD = oral liquid comparator used as standard treatment at time of study.

Study No.	Drug and Formulations	Participants *	Data Collected	Study Design
1	Midazolam (MDZ) as MDZ TMT MDZ LQD	Children aged 4–16 years scheduled for procedure and prescribed midazolam in hospital	Five-point hedonic scale to score liking for the taste; Answer Y/N to a question on willingness to take formulation again when sick	Randomised clinical trial; participant evaluated only one formulation, with administered midazolam dose based on body weight
2	Tramadol (TRM) as TRM TMT TRM LQD	Children aged 3–16 years scheduled for procedure and prescribed tramadol in hospital	Five-point hedonic scale to score liking for the taste; Answer Y/N to a question on willingness to take formulation again when sick	Randomised clinical trial; participant evaluated only one formulation, with administered tramadol dose based on body weight
3	Flucloxacillin (FLX) as FLX TMT1 FLX TMT2 FLX LQD	Healthy young adult volunteers aged 18–25 years	Five-point hedonic scale to score liking for the taste; Answer 1, 2, or 3 to a question on which formulation they were most willing to take when sick	Repeated measures preclinical study; participant evaluated all 3 formulations at fixed flucloxacillin dose, in assigned randomised order with washout period
4	Prednisolone sodium phosphate (PSP) as PSP TMT PSP LQD	Healthy young adult volunteers aged 18–25 years	Eleven-point scale to score liking for formulation; Eleven-point scale to score willingness to take formulation again if needed; Answer 1 or 2 to a question on which formulation they were more willing to take when sick	Repeated measures preclinical study; participant evaluated both formulations at fixed prednisolone sodium phosphate dose, in assigned randomised order with washout period

* In all studies, participants were excluded if they had known allergies; were unable to understand the trial information sheet or consent form; were taking medications known to interact with the study drugs; were pregnant; or had smoked within the 48 h prior. For Study 2, additional exclusion criteria included significant renal or hepatic impairment; a history of seizure disorder or epilepsy; recent surgery; removal of tonsils or adenoids; obesity, defined as a BMI above the 95th percentile; severe obstructive sleep apnoea confirmed by an overnight sleep study; or severe respiratory disease with a significant impact on daily life.

**Table 2:** The number of participants evaluating each test medicinal formulation and the number, with the percentage in parenthesis, expressing a willingness to retake the formulation if unwell. Tramadol = TRM; Midazolam = MDZ; Flucloxacillin = FLX; Taste-masked tablets = TMTs; LQD = Oral Liquid comparator. Two flucloxacillin taste-masked tablets, FLX TMT1 and FLX TMT2, were evaluated.

Medicinal Formulation	TRM TMT	MDZ TMT	FLX TMT1	FLX TMT2	TRM LQD	MDZ LQD	FLX LQD
Number of participants	68	74	21	21	71	70	21
Median taste score	4	3	4	3	2	1	2
Mean (SD) taste score	3.69 (1.38)	3.19 (1.43)	3.52 (1.03)	3.19 (1.29)	2.00 (1.19)	1.71 (1.13)	2.10 (1.18)
Number (%) of participants willing to retake the formulation when unwell	53 (78%)	45 (61%)	11 (52%)	9 (43%)	24 (34%)	26 (37%)	1 (5%)

**Table 3:** The comparison of Medicine Acceptability Scores (MASs) and Willingness to Take Medicine Score (WTMS) for 7 medicinal formulations evaluated in Studies 1–3. MAS was calculated using taste scores obtained via a 5-point hedonic scale and applying different passives score ranges. WTMS was calculated based on the percentage of affirmative responses to the question on how willing the participant was to take the formulation again when unwell. Tramadol = TRM; Midazolam = MDZ; Flucloxacillin = FLX; Taste-masked tablets = TMTs; LQD = Oral Liquid comparator. Two flucloxacillin taste-masked tablets, FLX TMT1 and FLX TMT2, were evaluated.

Passives Score Range Applied	MAS Calculated for Each Medicinal Formulation						
	TRM TMT	FLX TMT1	MDZ TMT	FLX TMT2	MDZ LQD	TRM LQD	FLX LQD
2–3	50	62	27	33	−50	−34	−24
2–4	28	14	0	0	−54	−41	−33
3	41	38	19	19	−74	−59	−57
3–4	19	−10	−8	−14	−79	−66	−67
WTMS	28	19	11	10	−13	−16	−28

**Table 4:** The number of participants evaluating each test medicinal formulation, along with the median, mean (SD), and number (%) of participants expressing willingness to take the formulation again if unwell. Prednisolone sodium phosphate = PSP; Taste-Masked tablet = TMT; LQD = Oral Liquid comparator.

Medicinal Formulation	PSP TMT	PSP LQD
Number of participants (n)	25	25
Median score for taste	7	5
Mean taste score (SD)	7.08 (2.40)	5.60 (2.33)
Median score for willingness to take again	8	6
Mean score (SD) for willingness to take again	7.32 (3.06)	6.44 (2.86)
Number (%) of participants showing a greater preference to take the formulation when unwell	14 (56%)	11 (44%)

**Table 5:** The comparison of Medicine Acceptability Scores (MASs) and Willingness to Take Medicine Score (WTMS) for two prednisolone sodium phosphate formulations evaluated in Study 4. Two MAS values were calculated, the first based on taste scores obtained via an 11-point scale and the second based on ‘willingness to retake’ scores obtained via a second 11-point scale. A passives score range of 5–7 was applied to calculate the two MASs. WTMS was calculated based on the percentage of affirmative responses provided by the participants when asked which one of the two formulations they were willing to take again when unwell. Prednisolone sodium phosphate = PSP; Taste-Masked tablet = TMT; LQD = Oral Liquid comparator.

Medicinal Formulation	PSP TMT	PSP LQD
MAS based on taste score	32	0
MAS based on willingness to retake formulation again	40	12
WTMS	6	−6

**Table 6:** The comparison of Medicine Acceptability Scores (MASs) and Willingness to Take Medicine Score (WTMS) for participants in the age groups ≤6 years and >6 years in Study 1 and Study 2. Study 1 evaluated two formulations of midazolam (MDZ), and Study 2 evaluated tramadol (TRM) formulations. All values were calculated based on taste scores obtained using a 5-point hedonic scale. A passive score range of 3 was applied to calculate the two MAS values. WTMS was calculated as the percentage of participants who responded affirmatively when asked if they would be willing to take the medicine again when unwell. TMT = Taste-Masked Tablet; LQD = Oral Liquid comparator.

Medicinal Formulation	Age of Participants	Number of Participants	MAS	WTMS
MDZ TMT	4–6	39	33.3	1.3
MDZ TMT	7–16	36	0.0	19.4
MDZ LQD	4–6	35	−60.0	−24.3
MDZ LQD	7–16	35	−88.6	−1.4
TRM TMT	3–6	32	56.3	28.1
TRM TMT	7–16	36	27.8	30.6
TRM LQD	3–6	32	−40.6	−15.6
TRM LQD	7–16	39	−74.4	−16.7

## Data Availability

The original contributions presented in the study are included in the article, further inquiries can be directed to the corresponding author due to ethical restrictions.
